# Overexpression of prostate tumor overexpressed 1 correlates with tumor progression and predicts poor prognosis in breast cancer

**DOI:** 10.1186/1471-2407-14-457

**Published:** 2014-06-19

**Authors:** Fangyong Lei, Longjuan Zhang, Xinghua Li, Xi Lin, Shu Wu, Fengyan Li, Junling Liu

**Affiliations:** 1 State Key Laboratory of Oncology in South China, Guangzhou 510060, China; 2 Laboratory of Surgery, First Affiliated Hospital, Sun Yat-sen University, No.58 Zhongshan 2nd Rd, Guangzhou 510080, China; 3 Ultrasonic department, Sun Yat-sen University Cancer Center, Guangzhou 510060, China; 4 State Key Laboratory of Oncology in South China and Department of Radiation Oncology, Sun Yat-sen University Cancer Center, Guangzhou 510060, China; 5 State Key Laboratory of Oncology in South China and Department of Medical Oncology, Sun Yat-sen University Cancer Center, Guangzhou 510060, China

**Keywords:** PTOV1, Breast cancer, Prognosis, Biomarker

## Abstract

**Background:**

Prostate tumor overexpressed 1 (PTOV1) was demonstrated to play an important role in cancer progression and was correlated with unfavorable clinical outcome. However, the clinical role of PTOV1 in cancer remains largely unknown. This study aimed to investigate the expression and clinicopathological significance of PTOV1 in breast cancer.

**Methods:**

The mRNA and protein expression levels of PTOV1 were analyzed in 12 breast cancer cell lines and eight paired breast cancer tumors by semi-quantitative real time-PCR and western blotting, respectively. Immunohistochemistry was performed to assess PTOV1 protein expression in 169 paraffin-embedded, archived breast cancer samples. Survival analysis and Cox regression analysis were performed to investigate the clinicopathological significance of PTOV1 expression.

**Results:**

Our data revealed that PTOV1 was frequently overexpressed in breast cancer cell lines compared to normal human breast epithelial cells and in primary breast cancer samples compared to adjacent noncancerous breast tissues, at both the mRNA and protein levels. Moreover, high expression of PTOV1 in breast cancer is strongly associated with clinicopathological characteristics and estrogen receptor expression status (*P* = 0.003). Breast cancer patients with higher PTOV1 expression had substantially shorter survival times than patients with lower PTOV1 expression (*P* < 0.001). Univariate and multivariate analysis revealed that PTOV1 might be an independent prognostic factor for breast cancer patients (*P* = 0.005).

**Conclusions:**

Our study showed that PTOV1 is upregulated in breast cancer cell lines and clinical samples, and its expression was positively associated with progression and aggressiveness of breast cancer, suggesting that PTOV1 could serve as an independent prognostic marker.

## Background

Human breast cancer is the most common carcinoma in females, and the second leading cause of cancer related mortality in women, accounting for approximately 29% (232,340) of all new cancer cases among women and 14% (39,620) cancer related mortality, representing a serious health threat to women worldwide [1,2]. Although various treatments for breast cancer, such as chemotherapy, radiation and hormone therapy, have been used and have been improved recently, the clinical outcome of patients remains unsatisfactory. This is largely because of a lack of effective and specific biomarkers that predict breast cancer. Thus, it is important to identify new genes and molecules that can effectively distinguish patients with favorable prognosis from those with poor prognosis, and to develop new therapy options for breast cancer patients.

Prostate tumor overexpressed 1 (PTOV1), a 46 kDa protein with two repeated PTOV homology blocks, was first identified during a screen for genes overexpressed in prostate cancer [3]. The PTOV1 gene is located on a region of chromosome 19 (19q13) that is associated with high risk of breast cancer [4,5]. PTOV1 comprises 12 exons, and the encoded protein has two almost identical tandemly arranged PTOV domains, each containing a potential nuclear localization signal [3]. PTOV1 expression is elevated in multiple cancers, including lung, endometrium, bladder, kidney and ovary cancer [6]. However, the expression and clinical relevance of PTOV1 in breast cancer have not been determined. Additionally, PTOV1 was reported to be associated with tumor development and progression. Recently, PTOV1 was shown to force cells to enter S phase and to promote mitotic activity of prostate cancer cells. High levels of PTOV1 expression are significantly associated with Ki67 immunostaining, indicating that PTOV1 upregulation is functionally related to proliferative status [7,8]. PTOV1 negatively regulates retinoic acid receptor transcription activity by antagonizing mediator complex subunit 25 [9]. Marqués N et al. reported that PTOV1 promotes c-Jun expression at the post-transcriptional level, which enhanced the invasive and metastatic capacity of prostate cancer cells [10]. Accumulating data indicate that PTOV1 might play an essential role in tumorigenesis.

In the present study, e aimed to investigate the expression of PTOV1 in breast cancer and its relationship with clinical parameters and prognosis in breast cancer patients. The results showed that PTOV1 is significantly upregulated in breast cancer, and overexpression of PTOV1 is closely associated with the clinical stage, T, N and M classification, and estrogen receptor (ER) expression levels in breast cancer. Cox regression analysis revealed that PTOV1 might be considered as an independent biomarker for breast cancer prognosis. Collectively, our findings strongly suggested that PTOV1 plays an important role in the development and progression of human breast cancer, and might be a useful predictive marker of prognosis in breast cancer patients.

## Methods

### Cell lines

Primary normal breast epithelial cells (NBEC) were established according to a previous report [11]. Immortalized breast epithelial cells MCF-01A were maintained in keratinocyte serum-free medium and breast cancer cell lines, including BT474, BT549, MDA-MB-435, MDA-MB-453, MDA-MB-231, MDA-MB-415, MDA-MB-468, T47D, MCF-7, ZR-75-1, ZR-75-30, SKBR-3, and Bcap-37 were purchased from ATCC and maintained in DMEM medium (Invitrogen) supplemented with 10% fetal bovine serum (HyClone, Logan, UT, USA) and 100 μg/ml penicillin, and 100 μg/ml streptomycin (Invitrogen) at 37°C in a humidified atmosphere containing 5% CO2.

### Tissue specimens and patient information

A total of 169 breast cancer paraffin-embedded specimens from female patients, which had been histopathologically and clinically diagnosed as breast cancer at the Cancer Center, Sun Yat-sen University from 2003 to 2007, were used in the present study. Tumor grade and stage were defined according to the 6th edition of the TNM classification of the Union for International Cancer Control (UICC, 2002). For the use of these clinical materials for research purposes, prior patients’ consents and approval from Sun Yat-sen University Cancer Center Institutional Review Board were obtained. Clinical information on the samples is summarized in Table 1. The median age at the time of surgery was 47 years (range 22–96 years). The follow-up time of the primary breast cancer cohort ranged from 9 to 115 months, and the median follow-up time was 59 months. The percentage of tumor purity in sections adjacent to the regions used for RNA extraction was estimated during routine histopathological analysis.

**Table 1:** Clinicopathological characteristics of patientsamples and expression of PTOV1 in breast cancer

**Variables**	**Number of cases (%)**
**Gender**	
Male	0 (0.0)
Female	169 (100.0)
**Age (years)**	
≥ 47	89 (52.7)
< 47	80 (47.3)
**Clinical stage**	
I	30 (17.8)
II	81 (47.9)
III	53 (31.4)
IV	5 (3.0)
**T classification**	
T_1_	52 (30.8)
T_2_	88 (52.1)
T_3_	17 (10.1)
T_4_	12 (7.1)
**N classification**	
N_0_	72 (42.6)
N_1_	50 (29.6)
N_2_	30 (17.8)
N_3_	17 (10.1)
**M classification**	
No	164 (97)
Yes	5 (3)
**Vital status (at follow-up)**	
alive	131 (77.5)
Dead	38 (22.5)
**Expression of PTOV1**	
Low expression	86 (50.9)
High expression	83 (49.1)
Detectable	167 (99.4)
Undetectable	2 (0.6)
**Expression of ER**	
0	74 (43.8)
1	37 (21.9)
2	16 (9.5)
3	42 (24.9)
**Expression of PR**	
0	75 (44.4)
1	28 (16.6)
2	27 (16.0)
3	39 (23.1)
**Expression of c-erbB2**	
0	90 (53.3)
1	20 (11.8)
2	13 (7.7)
3	46 (27.2)

### RNA extraction, reverse transcription and real-time PCR

Total RNA from cells and fresh surgically obtained tumor tissues and their adjacent noncancerous tissues was extracted using the Trizol reagent (Invitrogen, Carlsbad, CA) according to the manufacturer’s instruction. The extracted RNA was pretreated with RNase-free DNase, and 2 μg of RNA from each sample was used for cDNA synthesis primed with random hexamers. For PCR amplification of PTOV1 cDNA, an initial amplification using PTOV1 specific primers was done with a denaturation step at 95°C for 10 min, followed by 28 cycles of denaturation at 95°C for 60 s, primer annealing at 58°C for 30 s, and primer extension at 72°C for 30 s. Upon completion of the cycling steps, a final extension at 72°C for 5 minutes was done before the reaction was stored at 4°C. Real-time PCR was applied to measure the fold of increase of PTOV1 mRNA in each of the primary breast tumors relative to the paired normal breast tissue obtained from the same patient. Real-time PCR primers were designed using the Primer Express v 2.0 software (Applied Biosystems). The sequences of real-time PCR primers were: PTOV1 Forward: CGAGTACAGGAGCATGAGCA and Reverse: CTTCACCAACAGAGACTGCG; GAPDH Forward: GACTCATGACCACGTCCATGC and Reverse: AGAGGCAGGGATGATGTTCTG. Expression data were normalized to the geometric mean of housekeeping gene Glyceraldehyde-3-phosphate dehydrogenase (GAPDH) to control the variability in expression levels and analyzed using the 2^-△△Ct^ method described by the previous report [12], and all experiments were performed in triplicate.

### Western blotting

Cells were washed twice with ice-cold phosphate-buffered saline (PBS), then lysed on ice in radioimmune-precipitation assay (RIPA; Cell Signaling Technology, Danvers, MA) buffer containing complete protease inhibitor cocktail (Roche Applied Sciences, Mannheim, Germany) and heated for 5 min at 100°C. Fresh tissue samples were ground to powder in liquid nitrogen and lysed with SDS-PAGE sample buffer. Equal protein samples (30 μg) were separated on 10.5% SDS polyacrylamide gels and transferred to PVDF membranes (Immobilon P, Millipore, Bedford, MA). Membranes were blocked with 5% fat-free milk in Tris-buffered saline containing 0.1% Tween-20 (TBST) for 1 h at room temperature. Membranes were incubated with anti-PTOV1 antibody (1:100, Sigma, HPA051812) overnight at 4°C, and then with horseradish peroxidase-conjugated goat anti-rabbit IgG (Santa Cruz Biotechnology, SC-2004). To evaluate PTOV1 expression, enhanced chemiluminescence system (ECL) prime Western blotting detection reagent (Amersham) were used according to the manufacturer’s instructions. α-Tubulin (Sigma, Saint Louis, MO) was used as a loading control.

### Immunohistochemistry analysis

Immunohistochemical analysis was done to measure PTOV1 protein expression in 169 human breast cancer tissues. Briefly, paraffin embedded specimens were cut into 4 μm sections and baked at 60°C for 2 hours, followed by deparaffinized with xylenes and rehydrated. Antigenic retrieval was done by submerging the Sections into EDTA antigenic retrieval buffer and microwaving. The sections were then treated with 3% hydrogen peroxide in methanol to quench the endogenous peroxidase activity, followed by incubation with 1% bovine serum albumin to block the nonspecific binding. Sections were then incubated with anti-PTOV1 rabbit polyclonal antibody (1:50, Sigma, HPA051812) overnight at 4°C. For negative controls, the primary antibody was replaced by normal goat serum. After washing, the tissue sections were treated with biotinylated anti-rabbit secondary antibody (Abcam), followed by a further incubation with streptavidin-horseradish peroxidase complex (Abcam). The tissue sections were immersed in 3-amino-9-ethyl carbazole and counterstained with 10% Mayer’s hematoxylin, dehydrated and mounted in Crystal Mount.

The degree of immunostaining of the sections was viewed and scored separately by two independent investigators, who were blinded to the histopathologic features and patient data of the samples. The scores were determined by combining the proportion of positively stained tumor cells and the intensity of staining. Scores given by the two independent investigators were averaged for further comparative evaluation of the PTOV1 expression. The proportion of positively stained tumor cells was graded as follows: 1 (< 10% positive tumor cells), 2 (10-50% positive tumor cells), 3 (50-75% positive tumor cells), and 4 (> 75% positive tumor cells). The intensity of staining was recorded as following: 0 (no staining), 1 (weak staining, light yellow), 2 (moderate staining, yellowish brown), and 3 (strong staining, brown). The staining index was calculated as the product of the proportion of positive cells and the staining intensity score. Cutoff values to define the high and low expression of PTOV1 were chosen based on a measure of heterogeneity with the log-rank test statistics with respect to overall survival (OS). An optimal cut-off value was identified: a staining index score of greater or equal to 6 was used to define tumors with high PTOV1 expression and a score less than or equal to 4 indicated low PTOV1 expression.

### Statistical analysis

All statistical analyzes were carried out using the SPSS 16.0 statistical software packages. The chi-square test and Fisher’s exact test were used to analyze the correlation between PTOV1 expression and the clinicopathologic characteristics. Bivariate correlations between study variables were calculated by Spearman’s rank correlation coefficients. Survival curves were plotted using the Kaplan-Meier method and compared with the log-rank test. The significance of various variables for survival was analyzed by univariate and multivariate Cox regression analyzes. All reported *P*-values are two-sided. A *P*-value of less than 0.05 was considered statistically significant in all cases.

## Results

### PTOV1 is upregulated in breast cancer cell lines

**Figure 1:**
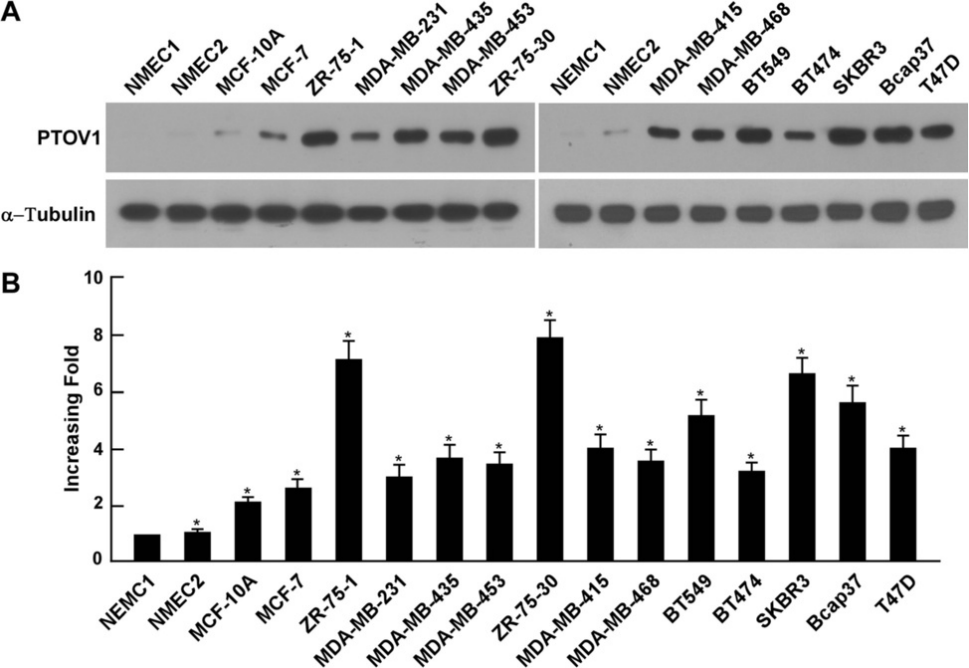
**Overexpression of PTOV1 in breast cancer cell lines and normal human mammary epithelial cell lines.** Expression of PTOV1 mRNA and protein in an immortalized “normal” breast epithelial cell line MCF-10A and breast cancer cell lines (MCF-7, ZR-75-1, MDA-MB-231, MDA-MB-435, MDA-MB-453, ZR-75-30, MDA-MB-415, MDA-MB-468, BT549, BT474, SKBR3, Bcap37, T47D) and two normal human mammary epithelial cell lines (NMEC1, NMEC2) were examined by western blotting **(A)** and real-time PCR **(B)**. Expression levels were normalized for α-Tubulin and GAPDH, respectively. Error bars represent SD from three independent experiments. *P < 0.05.

To explore the potential role of PTOV1 in the tumorigenesis of breast cancer, the expression of the PTOV1 protein and mRNA were determined by western blotting and real time-PCR. Higher PTOV1 protein expression was observed in all 12 breast cancer cell lines compared with that in two primary cultured normal human mammary epithelial cells (NMEC), in which it was weakly detected (Figure 1A). In line with the finding of protein expression, the mRNA expression of PTOV1 was significantly higher in breast cancer cell lines than in NMEC (Figure 1B). These results indicated that PTOV1 is upregulated in breast cancer cell lines. Since the cell lines we tested vary in ER status, PR status, HER2 status, mutant p53 status, and they belong to either luminal or basal-like type, we summarized these statuses and analyzed the correlation between PTOV1expression and these statuses (Additional file 1: Table S1). Briefly, we define the high or low PTOV1 expression by the median of the density scan of Western blotting detection of the protein expression of the cell lines. The results showed that there were no significant differences between PTOV1 expression and these features of the breast cancer cell lines. This may due to that backgrounds of these cell lines we used in the study are not well understood and these genes express differently in cell lines and breast cancer tissues.

### PTOV1 is overexpressed in breast cancer tissues and associated with breast cancer progression

**Figure 2:**
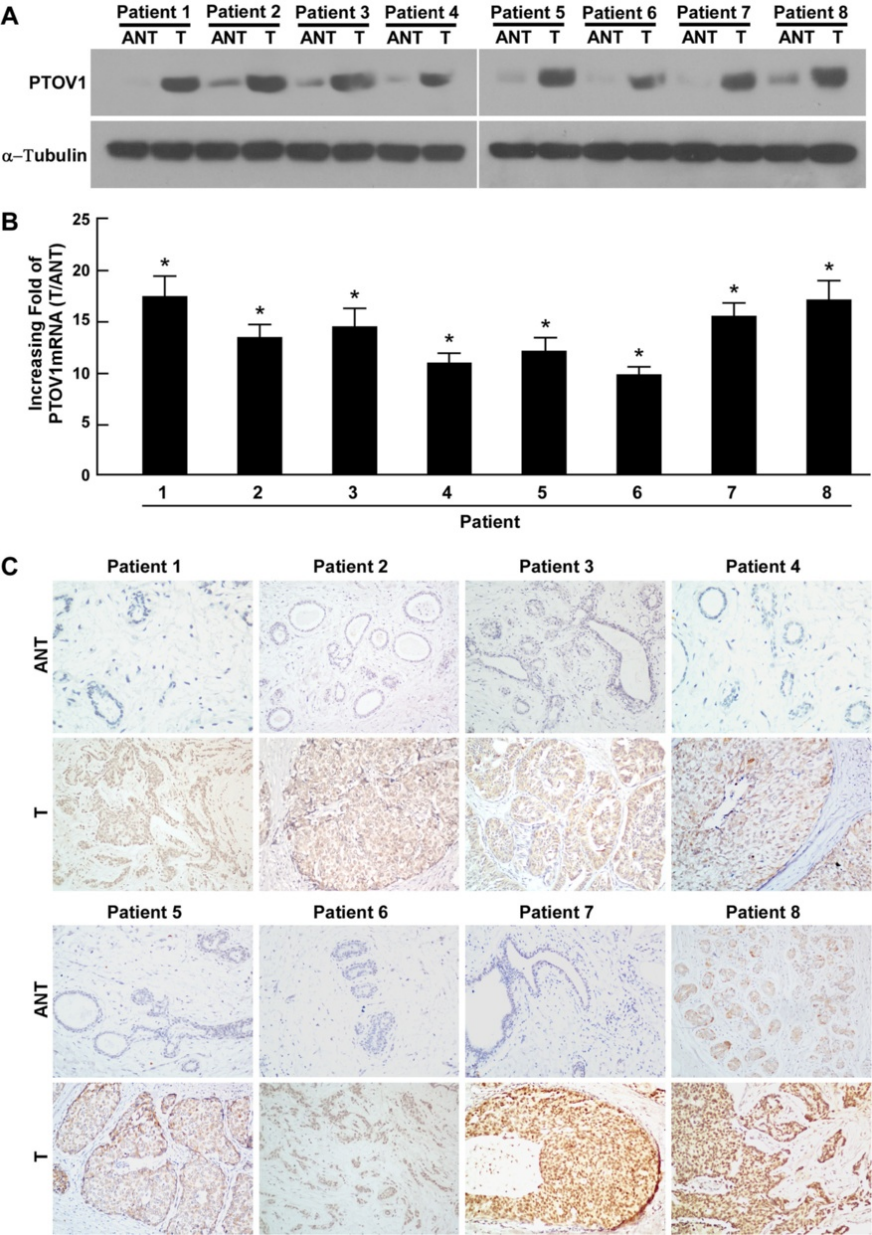
**Overexpression of PTOV1 in breast cancer tissues.** Expression of PTOV1 protein and mRNA in eight pairs of breast tissues (T) and matched adjacent non-cancerous tissues (ANT) from the same patient were determined by western blotting **(A)** and real-time PCR **(B)**, respectively. **(C)** Expression of PTOV1 protein in each of the primary breast cancer tissues and matched adjacent non-cancerous tissues examined by immunohistochemistry. *P < 0.05.

To determine whether PTOV1 is upregulated in breast cancer tissues, four pairs of matched breast cancer tissues and the noncancerous tissue adjacent to the malignant lesion were used for the further examination. As shown in Figure 2, PTOV1 was markedly upregulated at both the protein and mRNA levels in all eight human primary breast cancer samples compared with the matched adjacent noncancerous breast tissues. Differential expression, as measured by the T/ANT ratio of mRNA levels, ranged between 10.2 fold to 17.8 fold. Consistent with the above data, the expression of PTOV1 protein was elevated at least 2.3-fold higher in cancer tissues compared with their noncancerous counterparts. In summary, these data suggested that PTOV1 is overexpressed in breast cancer tissues.

**Figure 3:**
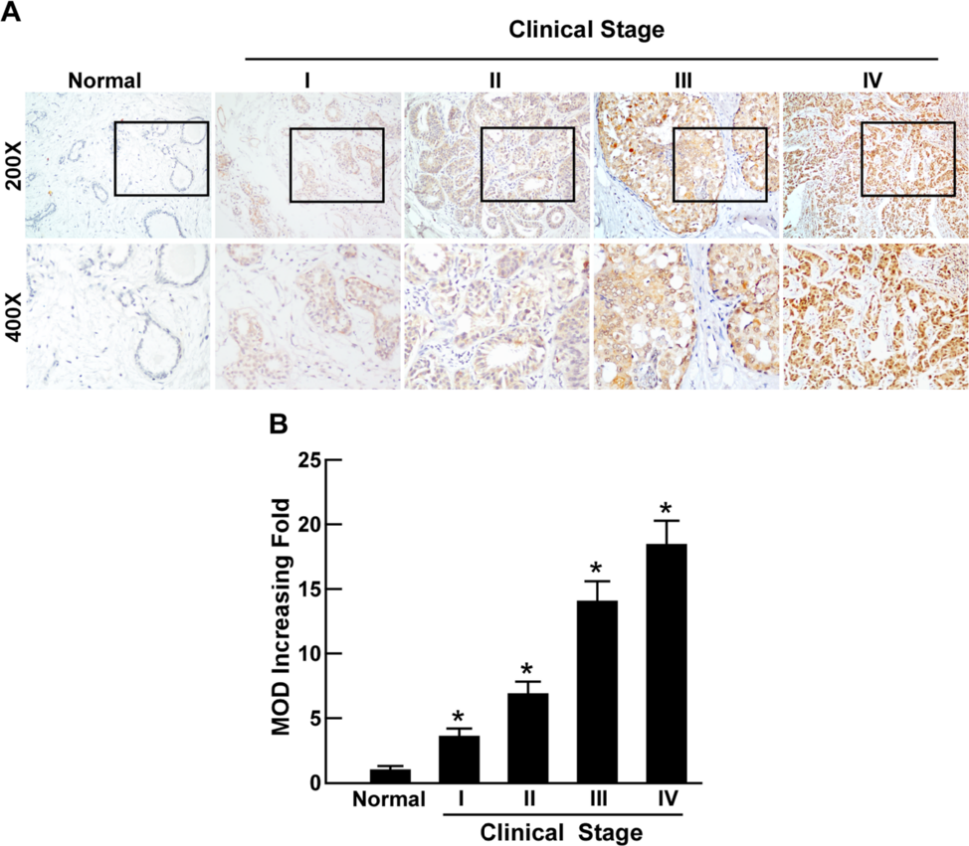
**PTOV1 protein overexpression in archived paraffin-embedded breast cancer tissue sections examined by immunohistochemistry. (A)** Representative IHC images of PTOV1 expression in normal human breast tissues and breast cancer tissues of different clinical stages. **(B)** Statistical analyses of the average MOD of PTOV1 staining between normal human breast tissues and breast cancer specimens of different clinical stages. *P < 0.05.

To further explore whether PTOV1 upregulation is associated with clinicopathological characteristics of breast cancer, we examined the PTOV1 expression status in 169 paraffin-embedded, archived breast cancer tissues by immunohistochemistry (IHC). The archived tissue came from 30 patients with stage I cancer, 81 patients at stage II, 53 patients at stage III, and five patients at stage IV. As shown in Table 1, PTOV1 expression was detected in 167 of 169 (99.4%) cases. Among the PTOV1-positive cases, 83 (49.1%) cases had high levels of PTOV1 expression, while 86 (50.9%) cases had low levels of PTOV1 expression. High PTOV1 expression was observed in areas containing primary breast cancer cells; however, it was undetectable or only marginally detectable in normal breast tissues and adjacent non-cancerous tissues (Figure 2). The subcellular location of PTOV1 was mainly in the cytoplasm of primary cancer cells, which is consistent with previous reports [8,12] (Figure 2). Furthermore, IHC revealed that increasing PTOV1 staining was positively correlated with advancing clinical stage and higher pathological grade (Figure 3A). Quantitative IHC analysis revealed that the median optical density (MOD) values of PTOV1 staining in all breast cancer were higher than that in control normal tissues. In addition, the MOD values of PTOV1 significantly increased with progression of tumor grades from I to IV (*P* < 0.001) (Figure 3B). Taken together, these results suggested that upregulation of PTOV1 is associated with breast cancer progression.

Statistical analysis was performed to evaluate the clinical significance between the PTOV1 expression and the clinicopathological parameters of breast cancer. As summarized in Table 2, PTOV1 expression was strongly correlated with clinical stage (*P* = 0.005), T classification (*P* = 0.048), N classification (*P* = 0.035), M classification (*P* = 0.021) and estrogen receptor (ER) expression levels (*P* = 0.003), but not significantly associated with age, progesterone receptor (PR) expression levels and c-erbB2 expression levels. Moreover, Spearman correlation analysis suggested that the PTOV1 expression level was markedly associated with clinical stage (r = 0.256, *P* = 0.001), T classification (r = 0.181, *P* = 0.018), N classification (r = 0.224, *P* = 0.003), M classification (r = 0.178, *P* = 0.021) and ER expression levels (r = 0.246, *P* = 0.001) (Table 3). Taken together, our data showed that overexpression of PTOV1 is positively correlated with clinical stage; T, N, M classification; and ER expression levels, which further supported the hypothesis that the elevated PTOV1 expression is associated with breast cancer progression.

**Table 2:** Clinicopathological characteristics of patient samples and expression of PTOV1 in breast cancer and correlation between PTOV1 expression and clinicopathological characteristics of breast cancer patients

**Characteristics**		**Total**	**PTOV1**		**chi-square test *P*-value**	**Fisher’s exact test *P*-value**
			**Low expression (%)**	**High expression (%)**		
Age (y)	≥ 47	82	43 (52.4)	39 (47.6)	0.695	0.406
	< 47	87	43 (49.4)	44 (50.6)		
Clinical stage	I	30	20 (66.7)	10 (33.3)	0.005	0.003
	II	81	46 (56.8)	35 (43.2)		
	III	53	20 (37.7)	33 (62.3)		
	IV	5	0 (0)	5 (100)		
T classification	T_1_	52	31 (59.6)	21 (40.4)	0.048	0.046
	T_2_	88	46 (52.3)	42 (47.7)		
	T_3_	17	7 (41.2)	10 (58.8)		
	T_4_	12	2 (16.7)	10 (83.3)		
N classification	N_0_	72	45 (62.5)	27 (37.5)	0.035	0.036
	N_1_	50	24 (48)	26 (52)		
	N_2_	30	12 (40)	18 (60)		
	N_3_	17	5 (29.4)	12 (70.6)		
M classification	Yes	5	0 (0.0)	5 (100.0)	0.021	0.027
	No	164	86 (52.4)	78 (47.6)		
Expression of ER	0	74	45 (60.8)	29 (39.2)	0.003	0.003
	1	37	23 (62.2)	14 (37.8)		
	2	16	5 (31.3)	11 (68.5)		
	3	42	13 (31.0)	29 (69.0)		
Expression of PR	0	75	42 (56.0)	33 (44.0)	0.092	0.093
	1	28	15 (53.6)	13 (46.4)		
	2	27	16 (59.3)	11 (40.7)		
	3	39	13 (33.3)	26 (66.7)		
Expression of HER 2	0	90	42 (46.7)	48 (53.3)	0.588	0.599
	1	20	12 (60.0)	8 (40.0)		
	2	13	8 (61.5)	5 (38.5)		
	3	46	24 (62.5)	22 (37.5)		
Vital status	Live	131	78 (59.5)	53 (40.5)	< 0.001	< 0.001
	Dead	38	8 (21.1)	30 (78.9)		

**Table 3:** Spearman correlation analysis between PTOV1 and clinical pathologic factors

**Variables**	**PTOV1 expression**	
	**Spearman correlation**	***p*-Value**
Clinical staging	0.256	0.001
T classification	0.181	0.018
N classification	0.224	0.003
M classification	0.178	0.021
ER expression	0.246	0.001
PR expression	0.141	0.067
HER2 expression	-0.069	0.372

### PTOV1 expression level is associated with the patient survival and prognosis

**Figure 4:**
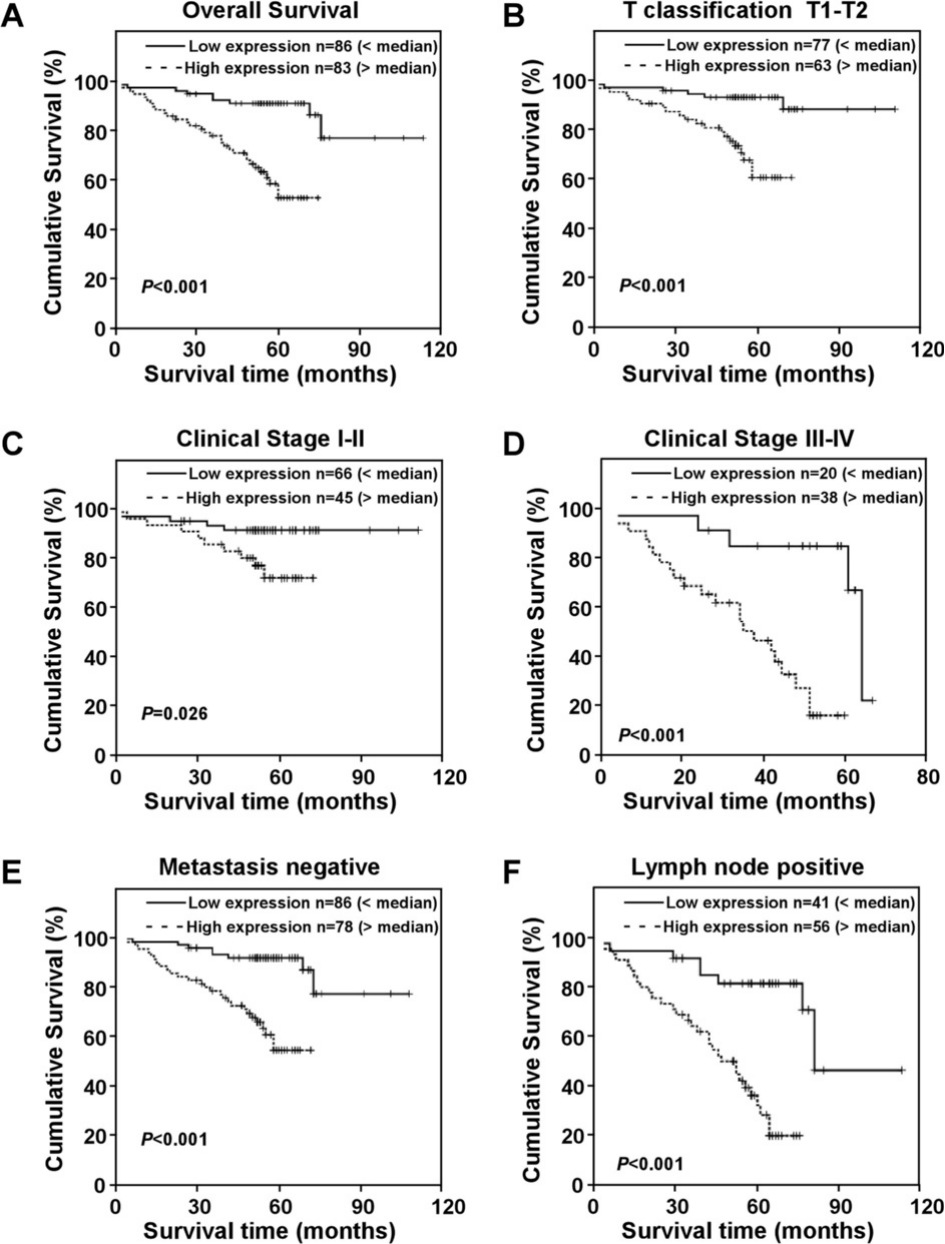
**Kaplan-Meier analysis of overall survival in breast patients based on PTOV1 expression. (A)** OS of all patients with high PTOV1 expression versus low PTOV1 expression. **(B)** OS rate for patients with high PTOV1 expression versus patients with low PTOV1 expression in patients with T1-T2 grade tumors. The overall survival of patients of high PTOV1 expression and low PTOV1 expression compared between patients with early stage disease (stage I–II) **(C)** and late stage disease (stage III–IV) **(D)** and between patients with negative metastasis disease **(E)** and between patients with positive lymph node metastasis **(F)**. P values were calculated by using log-rank tests.

Assessment of patient survival by the Kaplan–Meier analysis indicated an adverse correlation between PTOV1 expression and overall survival time of patients with breast cancer (*P* < 0.001) (Figure 4A). The 5-year cumulative survival rates of patients with higher PTOV1 expression and lower PTOV1 expression were 66.3% and 91.6%, respectively. The median survival time of patients with breast cancer with low PTOV1 expression was 115 months, compared with 78 months for patients with high PTOV1 expression.

Univariate and multivariate Cox proportional hazard regression analysis were performed to identify the independent prognostic value of each variable for predicting overall survival in patients. PTOV1 expression (*P* = 0.008) and clinicopathological characteristics: pT status (*P* = 0.006), pN status (*P* = 0.001) and PR expression (*P* = 0.017), which showed significant effects on overall survival by univariate analysis, were included in multivariate analysis (Table 4). As expected, PTOV1 was identified as an independent prognostic factor for patients with poor survival (relative risk: 1.230, 95% CI: 1.475-8.025, *P* = 0.005).

**Table 4:** Univariate and multivariate analyses of various prognostic parameters in patients with breast cancer Cox-regression analysis

**Variables**	**Univariate analysis**			**Multivariate analysis**		
	**No. patients**	***p***	**Regression coefficient (SE)**	***p***	**Relative risk**	**95% confidence interval**
**PTOV1**		0.008	0.571 (0.206)	0.005	1.230	1.457-8.025
Low expression	86					
High expression	83					
**T classification**		0.006	0.429 (0.214)	0.003	0.507	1.181-2.327
1	52					
2	88					
3	17					
4	12					
**N classification**		0.001	0.970 (0.263)	<0.001	0.808	1.625-3.097
0	72					
1	50					
2	30					
3	17					
**PR expression ssstas**		0.017	0.345 (1.412)	0.012	0.340	1.079-1.820
0	75					
1	28					
2	27					
3	39					

Moreover, the prognostic value of PTOV1 expression was analyzed when stratifying the patients according to different pT/pN/pM statuses and clinical stages. These findings suggested that overexpression of PTOV1 was an strong inverse prognostic factor for breast cancer patients in clinical stage I–II (early stage, *P* = 0.026) and III–IV (late stage, *P* = 0.001), indicating that PTOV1 could be considered as a valuable prognostic marker for breast cancer in all disease stages (Figure 4B, C). Similarly, patients with higher PTOV1 expression level had significantly shorter survival time in groups of: pT1-2 (*P* < 0.001) (Figure 4D), lymph node metastasis positive (including: pN1, pN2 and pN3) (*P* < 0.001) (Figure 4E) and pM0 (*P* < 0.001) (Figure 4F). However, no statistically significant differences were identified between PTOV1 expression and survival time in subsets of pT3-4, pN0 and pM1, which might reflect the limited number of patients recruited in each subset. Taken as a whole, these results indicate that PTOV1 could be a useful prognostic factor in breast cancer patients.

## Discussion

In the present study, we provided the first evidence that overexpression of PTOV1 protein is associated with poor prognosis of breast cancer patients with both early- and late-stage disease. Our data showed that PTOV1 is upregulated in breast cancer cell lines and in clinical tumor specimens, at both the mRNA and protein levels, compared with normal breast epithelial cells and normal breast tissues, respectively. Moreover, the analysis of 169 archived breast cancer samples revealed that PTOV1 expression is significantly associated with progression of breast cancer; a high level of PTOV1 might correlate with a shorter survival time (*P* < 0.001), indicating that PTOV1 plays an important role in breast cancer progression. Furthermore, Cox regression analysis showed that higher PTOV1 expression was an independent prognostic indicator of shorter survival in breast cancer patients. These findings strongly suggested that lower expression of PTOV1 would provide a selective advantage in prognosis for breast cancer patients.

PTOV1 was first identified as a novel elevated expressed gene in prostate cancer [3]. Aberrant PTOV1 expression is associated with tumor progression in prostate cancer and other neoplasms [3,6]. PTOV1 was reported to assist Flotillin-1 nuclear translocation and promote the mitogenic activity of Flotillin-1 in a cell cycle-dependent manner [7]. Flotillin-1 promotes tumorigenesis of various cancers, such as esophageal squamous cell carcinoma, non-small cell lung cancer and breast cancer [13-16]. Flotillin-1 promotes breast cancer carcinogenesis inducing entry into the S phase of the cell cycle and by upregulating the transcription factor Foxo3a [16]. Thus, we assumed that PTOV1 might promote breast cancer progression through a similar mechanism. Further investigations are required to test this assumption. In quiescent cells, PTOV1 is mainly localized in the cytoplasm and is excluded from the nucleus. However, elevated nuclear PTOV1 is closely correlated with high Ki67 immunoreactivity, indicating that PTOV1 plays an important role in cells proliferative status [8]. In xenograft experiments, tumor cells overexpressing PTOV1 showed an increased growth rate and tumorigenic capacity [10]. Taken together, these results confirmed that overexpression of PTOV1 could contribute to the proliferative status of tumor cells, indicating that PTOV1 is involved in the progression of cancer.

Recently, PTOV1, which is a modulator of the mediator transcriptional regulatory complex, was revealed to regulate c-Jun expression at the posttranscriptional level [10]. c-Jun is a major component of the AP-1 complex and is associated with a variety of biological processes, including proliferation and differentiation [17]. c-Jun is thought to induce mammary cell invasiveness, which plays an important role in breast cancer metastasis and stem cell expansion [18]. Therefore, we speculated that PTOV1 might promote breast cancer tumorigenesis through the transcription factor c-Jun and its downstream genes. Furthermore, a previous study found that high PTOV1 expression might be a good predictor of prostate cancer in men with isolated high-grade prostatic intraepithelial neoplasms in needle biopsies [19,20].Thus, PTOV1 might be a positive regulator of breast cancer development and progression; however, the exact mechanism needs further investigation.

Breast cancer patients with the same clinical stage, which is routinely classified by TNM stage system, often have distinct outcomes. This large difference indicates that the TNM stage system alone is not sufficient to fully predict the clinical outcome of breast cancer patients with regard to the heterogenetic biological characteristic of this malignancy. The ER is overexpressed in about 50% breast cancer patients and could be a prognostic indicator for breast cancer patients [21]. However, there is dispute over the definition of ER positive status nuclear staining by the routinely used IHC method, which has limited its clinical application [22-24]. Moreover, not all ER positive breast cancer patients respond to endocrine therapy; some of the patients who were sensitive to hormone therapy developed resistance; however, the mechanism is unclear [25,26]. Collectively, these defects limit the application of ER status in breast cancer management. A close correlation between PTOV1 expression and ER expression status (r = 0.246, *P* = 0.001) suggested that the expression level of PTOV1 might be a useful supplement to breast cancer hormone therapy decision-making. Thus, PTOV1 may be a useful marker for determining prognosis and guiding the follow-up schedule of breast cancer patients.

Our study indicated that PTOV1 might positively regulate breast cancer development and progression, and is a useful indicator of poor prognosis and a prognostic marker for patient survival. However, the modulation of PTOV1 expression in this malignant tumor and its molecular mechanisms in breast cancer development and progression still require further investigation.

## Conclusions

In this study, we found that PTOV1 overexpression is correlated with breast cancer progression and progressive phenotype. Moreover, our data indicated that PTOV1 might be an independent prognostic marker on the whole group and subgroup analysis as well. Thus, PTOV1 protein expression might be a useful marker for stratifying breast cancer patientsprognosis as well as an effective novel criteria for selection of therapeutic options.

## Competing interests

The authors declare that they have no competing interests.

## Authors’ contributions

FYLei carried out the Western blotting, and drafted the manuscript. LJZ collected the tissue specimens and patient information, and editing of the manuscript. XHL collected patient information and carried out the statistical analyses. XL carried out Immunohistochemical (IHC) analysis. SW carried out RNA extraction and real-time PCR. FYLi participated in designing the study and guiding editing the manuscript. JLL conceived the study and guided editing manuscript. All authors read and approved the final manuscript.

## Pre-publication history

The pre-publication history for this paper can be accessed here:

http://www.biomedcentral.com/1471-2407/14/457/prepub

## Supplementary Material

Additional file 1: Table S1 Subtype classification and ER, PR, HER2 and P53 status in breast cancer cell lines.doc. Click here for file
