# Supplementary material for: Overexpression of prostate tumor overexpressed 1 correlates with tumor progression and predicts poor prognosis in breast cancer
Source: BMC Cancer. 2014 Jun 19;14:457. doi: 10.1186/1471-2407-14-457 (PMC4070404; doi:10.1186/1471-2407-14-457)
Supplement: Additional file 1: Table S1 — Subtype classification and ER, PR, HER2 and P53 status in breast cancer cell lines.doc. [file 1471-2407-14-457-S1.doc]

**Additional files**

**Supplemental Table 1: Subtype classification and ER, PR, HER2 and P53 status in breast cancer cell lines.doc**

**Supplemental Table 1 Subtype classification and ER, PR, HER2 and P53 status in breast cancer cell lines**

| Breast cancer  cell line | Subtype | ER | PR | HER2 | P53 | PTOV1  expression |
| --- | --- | --- | --- | --- | --- | --- |
| MCF-7 | Luminal^[1,2,6]^;  luminal A^[3,4]^ | +^[3,5,6]^ | +^[3,5,6]^ | +/-^[3]^;-^[6]^ | WT^[5,7]^ | High |
| MDA-MB-231 | Basal^[2,3,6]^;  BasalB^[1]^ | -^[3,5,6]^ | -^[3,5,6]^ | +/-^[3]^;- ^[5,6]^ | Mutant^[5,7]^ | Low |
| MDA-MB-435 | Basal^[1]^;  HER2^[2]^ | -^[3]^ | -^[3]^ | +^[3]^ | NA | Low |
| MDA-MB-453 | Luminal^[1,2,6]^;  unclassified^[3]^;  HER2^[4]^ | -^[3,5,6]^ | -^[3,5]^;+ ^[6]^ | -^[3,5]^;+ ^[6]^ | WT^[5]^;  Deletion^[7]^ | Low |
| MDA-MB-468 | Basal^[2,3,4]^;  Basal A^[1,6]^ | -^[6]^ | -^[6]^ | -^[6]^ | Mutant^[5,7]^ | High |
| BT474 | Luminal^[1,2,6]^;  Luminal B^[3,4]^ | -^[3,5]^;+ ^[6]^ | +^[3,6]^;- ^[5]^ | +^[3,5,6]^ | Mutant^[5,7]^ | Low |
| BT549 | Basal^[2,6]^ | -^[5,6]^ | -^[5,6]^ | -^[5,6]^ | Mutant^[5,7]^ | High |
| T47D | Luminal^[l,2,6]^ ;  Luminal A^[4]^ | +^[5,6]^ | +^[5,6]^ | -^[6]^ | Mutant^[5]^;  WT^[7]^ | High |
| ZR-75-1 | Luminal^[1,2,3,6]^ | +^[3,5,6]^ | +^[3,5]^;- ^[6]^ | +^[3]^;- ^[5,6]^ | WT^[5,7]^ | Low |
| ZR-75-30 | Luminal^[3,6]^ | +^[6]^ | -^[6]^ | +^[5,6]^ | WT^[5]^ | High |
| SKBR3 | Luminal^[1,2,6]^;  HER2^[3,4]^ | -^[6]^ | -^[6]^ | +^[5,6]^ | WT^[5]^ | High |
| MCF-10A | Basal^[2,3,6]^ | -^[3,6]^ | -^[3,6]^ | +/-^[3]^;- ^[6]^ | NA | Low |
| MDA-MB-415 | Luminal^[2]^ | +^[5]^ | +^[5]^ | NA | Mutant^[5,7]^ | High |
| Bcape37 | NA | NA | NA | NA | NA | Low |

*NA: Unknown. There’s no research report about subtype classification or ER, PR, HER2, P53 status about the breast cancer cell line.

**Supplemental references:**

1. Venkata Lokesh Battula, Yuexi Shi, Kurt W. Evans, Rui-Yu Wang, Erika L. Spaeth, Rodrigo O. Jacamo, Rudy Guerra, Aysegul A. Sahin, Frank C. Marini, Gabriel Hortobagyi, Sendurai A. Mani, and Michael Andreeff: **Ganglioside GD2 identifies breast cancer stem cells and promotes tumorigenesis**. *J Clin Invest*. 2012 Jun 1; **122 (6)**: 2066-78

2. Lauren L.C. Marotta, Vanessa Almendro, Andriy Marusyk, Michail Shipitsin, Janina Schemme, Sarah R. Walker, Noga Bloushtain-Qimron, Jessica J. Kim, Sibgat A. Choudhury, Reo Maruyama, Zhenhua Wu, Mithat Gönen, Laura A. Mulvey, Marina O. Bessarabova, Sung Jin Huh, Serena J. Silver, So Young Kim, So Yeon Park, Hee Eun Lee, Karen S. Anderson, Andrea L. Richardson, Tatiana Nikolskaya, Yuri Nikolsky, X. Shirley Liu, David E. Root, William C. Hahn, David A. Frank, and Kornelia Polyak: **The JAK2/STAT3 signaling pathway is required for growth of CD44+CD24– stem cell–like breast cancer cells in human tumors**. *J Clin Invest*. 2011 Jul; **121 (7)**: 2723-35.

3. Kristina Subik, Jin-Feng Lee, Laurie Baxter, Tamera Strzepek, Dawn Costello, Patti Crowley, Lianping Xing, Mien-Chie Hung, Thomas Bonfiglio, David G. Hicks and Ping Tang: **The Expression Patterns of ER, PR, HER2, CK5/6, EGFR, Ki-67 and AR by Immunohistochemical Analysis in Breast Cancer Cell Lines**. *Breast Cancer (Auckl)*. 2010 May 20; **4**:35-41.

4. Deborah L Holliday and Valerie Speirs: **Choosing the right cell line for breast cancer research.** *Breast Cancer Res*. 2011 Aug 12; **13 (4)**: 215.

5. Xiaolan Hu, Howard M. Stern, Lin Ge, Carol O'Brien, Lauren Haydu, Cynthia D. Honchell, Peter M. Haverty, Brock A. Peters, Thomas D. Wu, Lukas C. Amler, John Chant, David Stokoe, Mark R. Lackner, and Guy Cavet: **Genetic alterations and oncogenic pathways associated with breast cancer subtypes**. *Mol Cancer Res*. 2009 Apr; **7 (4)**: 511-22.

6. Jessica Kao1, Keyan Salari, Melanie Bocanegra, Yoon-La Choi, Luc Girard, Jeet Gandhi, Kevin A. Kwei, Tina Hernandez-Boussard, Pei Wang, Adi F. Gazdar, John D. Minna, Jonathan R. Pollack: **Molecular Profiling of Breast Cancer Cell Lines Defines Relevant Tumor Models and Provides a Resource for Cancer Gene Discovery**. *PLoS ONE* **4 (7)**: e6146

7. Marijke Wasielewski, Fons Elstrodt, Jan G.M. Klijn, Els M.J.J. Berns, and Mieke Schutte: **Thirteen new p53 gene mutants identified among 41 human breast cancer cell lines**. *Breast Cancer Res Treat*. 2006 Sep; **99 (1)**: 97-101.
